# Perceived roles and barriers in caring for the people who are homeless: a survey of UK community pharmacists

**DOI:** 10.1007/s11096-019-00789-4

**Published:** 2019-01-18

**Authors:** Vibhu Paudyal, Kathrine Gibson Smith, Katie MacLure, Katrina Forbes-McKay, Andrew Radley, Derek Stewart

**Affiliations:** 1 School of Pharmacy, University of Birmingham, Birmingham, B15 2TT UK; 2 School of Pharmacy and Life Sciences, Robert Gordon University, Aberdeen, UK; 3 School of Social Studies, Robert Gordon University, Aberdeen, UK; 4 NHS Tayside, Dundee, UK

**Keywords:** Community pharmacist, Community pharmacy, Counselling, Homeless, Signposting, United Kingdom

## Abstract

*Background* Community pharmacists can be an accessible source for advice and support for the people who are homeless, given their utilisation of a variety of currently available services such as dispensing of medicines, drugs and alcohol services. *Objective* To determine community pharmacists’ training, experiences and behavioural determinants in counselling and management of homeless population. *Setting* UK community pharmacies. *Method* A questionnaire based on literature and theoretical domains framework was mailed to randomly sampled community pharmacies in England and Scotland (n = 2000). Data were analysed using descriptive and inferential statistics. *Main outcome measures* Pharmacists’ perspectives, pharmacists’ training, pharmacists’ experiences and behavioural determinants. *Results* A total of 321 responses (RR 16.1%) were received. Respondents indicated lack of knowledge, skills, intentions as well as contextual factors such as lack of guidelines impacted on their counselling and management of homeless patients. Less than a third (n = 101, 32.2%) indicated that they knew where to refer a homeless patient for social support. Broaching the subject of homelessness was outside their comfort zone (n = 139, 44.3%). Only four (1.2%) respondents could correctly answer all knowledge assessment questions. *Conclusions* Community pharmacist identified lack of education, training opportunities and guidelines in counselling and management of homeless patients. Targeting community pharmacists’ knowledge, skills and intention to provide care to the homeless patients may enable addressing health inequality through community pharmacy.

## Impacts on practice

- Community pharmacists can benefit from appropriate training and guidelines in offering proactive support and advice to homeless patients.
- Establishing appropriate guidelines will enable community pharmacists to be aware of their remit in advising homeless patients on wider aspects of health and social care.
- Incorporating homelessness in undergraduate education and professional development training can improve pharmacists’ knowledge and confidence in caring for the people who are homeless.

## Introduction

The term homelessness extends beyond merely rough sleeping and encompasses living in derelict buildings, temporary shelters, squats or sofa surfing [1]. Homelessness is a pertinent issue both in the United Kingdom (UK) and worldwide [2]. A 10% reduction in real income for typical working families was observed in 2016/2017 when compared to 2008 levels [3]. In addition, there has been an estimated 37% real-term reduction in government funding to local authorities in the UK from 2010 to 2016 [4]. Economic austerity has hence been blamed on rising homelessness, particularly in urban areas. Nearly twice as many people sleep rough on any given night now in England than in 2010 [5, 6].

Those experiencing homelessness are significantly disadvantaged in attaining and maintaining a healthy lifestyle [7–10]. Injury, assault and skin problems are commonly experienced amongst those who are sleeping rough with health status worsening as homelessness persists. The homeless population has a higher rate of mortality than the general population with street dwellers and those occupying homeless shelters dying at an average age of 47 years [11]. Opioid over-dose, accidents, heart failure and infectious diseases contribute to higher mortality rates [12–14]. Addressing health inequality requires specific focus on disadvantaged populations.

Pharmacists in the UK offer services often utilised by homeless persons including dispensing of medicines, opioid replacement therapy (ORT) and needle exchange services. Recent published strategic Government approaches in the UK advise that the pharmacy workforce increases their role in promoting health in an effort to enhance both capacity and capability in reducing health inequality including amongst vulnerable groups [15–17].

With a greater policy emphasis on integrated working across health and social care, there are greater expectations and opportunity for joined-up working across sectors. Pharmacists, due to their day-to-day patient facing roles, are potentially suited to working more collaboratively with social care services. Collaborative work may include making referrals to wider services available in the community such as social housing, primary care services and free meals. For example, methadone services are usually offered on a daily dispense schedule from community pharmacy. This allows pharmacists to offer opportunistic healthcare interventions and signpost to appropriate social care support and services.

There have been recent examples of pharmacies’ widening participation in their role to alleviate the health impact of homelessness in the UK. A service model whereby homeless persons consulted with community pharmacists was recently piloted in Glasgow and demonstrated the wide ranging services provided including prescribing, diagnostic and referrals to specialist clinics [18]. Our previous qualitative research with the homeless population has demonstrated under-utilisation of pharmacy services advice on health, medicines related and social care signposting related queries [19, 20]. Respondents reported experiencing lack of means to access pharmacy such as bus fares and perceived discrimination by members of pharmacy staff and the general public in pharmacy premises. This may have discouraged some patients from accessing pharmacy services. Lack of secure places for storing medicines, sharing and theft of medicines were key barriers in maintaining adherence to prescribed medicines. The results suggest that there may be new roles for community pharmacists in supporting homeless patients. Having appropriate skills, knowledge and training is imperative to pharmacists providing relevant support, information and advice to people who are homeless who have unique and specific needs. There is a dearth of research exploring community pharmacists’ role in offering wider forms of support including signposting to the people who are homeless.

For the purpose of the research, and as indicated in the mailed questionnaire, the term ‘managing homeless patients’ referred to advice giving, dispensing of prescribed medicines, referral or any other professional activity in relation to the care of homeless patients.

## Aim of the study

This study aimed to determine pharmacists’ training, experiences, current roles and barriers in counselling and management of homeless patients.

## Ethics approval

This study was approved by the Robert University Research Ethics Committee. The NHS Research Ethics Committee advised that a full NHS Ethics submission was not required. Health Research Authority (England) and NHS Research and Development (Scotland) approvals were obtained prior to commencing the study.

## Method

A cross sectional survey design in the form of a mailed questionnaire was utilised. The questionnaire was based on existing literature, the previous research [19–22] and expert opinion amongst the research team. The theoretical domains framework (TDF) (version 2) was used to construct questionnaire items to identify pharmacists’ behaviours and beliefs (Table 2). The TDF is a theoretical framework which synthesises 33 theories of behaviour change into 14 domains [23]. Eleven of the 14 TDF domains were used in the questionnaire development. The questionnaire consisted of both closed (including Likert-type attitudinal and agreement scales) and open ended questions. Respondents were also presented with a multiple choice quiz (MCQ) to determine their knowledge of homelessness.

The questionnaire was reviewed for face and content validity by an expert panel (one GP, one nurse practitioner and one community pharmacist), and piloted with 50 pharmacists to determine the response rate and questionnaire suitability. In addition, the use of literature, theory and researcher expertise added to the validation exercise.

A list of pharmacy premises in England and Scotland was obtained from the General Pharmaceutical Council (GPhC), the regulatory body overseeing the profession of pharmacy in the UK [24]. Questionnaire was sent to a random sample of 2000 (of the approximate total of 12,900) community pharmacies across England and Scotland in November 2016–March 2017 addressing ‘the responsible pharmacist’ [25]. The random sample was generated using SPSS V.24. Two reminders were sent at 2 week intervals. Return of a completed questionnaire implied consent to participate.

### Data management and analysis

Data were analysed using descriptive statistics including frequencies and percentages. Reliability analysis was undertaken on the scale items as per the TDF. Scales were considered reliable based on Cronbach’s alpha value ≥ 0.7. Scale means, variance and standard deviations were also extracted. The Scottish Index of Multiple Deprivation [26] along with the English Indices of Deprivation [27] were used to generate deprivation indexes from the postcodes provided of pharmacy (Table 1). Separate classification method of deprivation exist in England and Scotland. Structured open ended questions were analysed through content analysis as per the theme of the questions presented to the participants.

**Table 1:** Respondent demography

Demographic characteristics	Number of respondents, n (%)
Sex (n = 317)	
Female	157 (49.5)
Male	155 (48.9)
Prefer not to say	5 (1.6)
Experience of work as a pharmacist (years) (n = 317)	
5 or less	94 (29.7)
6–10	50 (15.8)
11–15	41 (12.9)
16–20	34 (10.7)
21–25	19 (6.0)
26–30	27 (8.5)
31 or more	51 (16.1)
Multiple deprivation index quintile (England) (n = 186)	
1 (least deprived)	29 (15.6)
2	33 (17.7)
3	36 (19.4)
4	34 (18.3)
5 (most deprived)	54 (29.0)
Multiple deprivation index quintile (Scotland) (n = 34)	
1 (most deprived)	12 (35.3)
2	7 (20.6)
3	6 (17.6)
4	7 (20.6)
5 (least deprived)	2 (5.9)
Is community pharmacy your main practice setting? (n = 317)	
Yes	311 (98.1)
No	6 (1.9)
Are you an independent prescriber? (n = 315)	
Yes	17 (5.4)
No	298 (94.6)
Do you have a postgraduate qualification? (n = 315)	
Yes	111 (35.2)
No	204 (64.8)
Services offered to the homeless^a^	
Dispensing or prescribing of medicines (n = 266)	166 (62.4)
Chronic medication service (n = 265)	3 (1.1)
Medicines use reviews (n = 266)	30 (11.3)
Minor ailments service (n = 265)	64 (24.2)
Opioid replacement therapy (n = 322)	155 (58.3)
Social support (n = 265)	21 (7.9)
Alcohol misuse (n = 266)	46 (17.3)
Promoting self-care (n = 265)	62 (23.4)
Other (n = 263)	39 (14.8)

^a^Response options included yes or no and multiple choices were allowed

## Results

A total of 322/1951 (16.1%) responses were received, of which 157 (49.5%) were female. Mean age was 39 years (range 22–69, SD 12.0). Over a third (n = 111, 35.2%) had a postgraduate qualification. Of the 322 respondents, 220 respondents (68.3%) provided post code data. Fifty-four (29.0%) and 12 (35.3%) pharmacies were located in the most deprived quintile in England and Scotland (Table 1).

### Experiences of managing homeless patients

Respondent experiences of managing homeless patients varied. Over a third of the respondents stated that they managed homeless patients either daily (n = 53, 16.7%), weekly (n = 33, 10.4%). Respondents reported managing between 0 and 50 homeless patients a month.

Those managing homeless reported that they offered a variety of services which may be utilised by those experiencing homelessness. The majority of respondents (n = 166, 62.4%) reported dispensing or prescribing medicines as being the most commonly delivered service to people who are homeless, followed by opioid replacement therapy (n = 155, 58.3%) (Table 2). Respondents reported provision of other services including acting as a contact point for key workers in the social care team, blood pressure and diabetes check, services offered to prisoners which included people had faced homelessness, needle exchange, new medicines services, in addition to signposting to dental services and general practitioners (GPs).

**Table 2:** TDF domains, description, questionnaire scale items and responses

TDF domains	Description (24)	Statements in the survey (n)	Responses n(%)					Cronbach’s Alpha^a^	Scale mean	Scale variance	SD
			Strongly disagree	Disagree	Neither agree nor disagree	Agree	Strongly agree				
Knowledge- self	An awareness of the existence of something	I know how homelessness affects patient health and wellbeing (315)	13 (4.1)	20 (6.3)	32 (10.2)	159 (50.5)	91 (28.9)	0.80	13.33	10.66	3.27
		I know how homelessness affects patient adherence to their medicines (315)	11 (3.5)	27 (8.6)	39 (12.4)	146 (46.3)	92 (29.2)				
		I know how to advise homeless patients about minimising the impact of homelessness on their use of medicines (314)	25 (8.0)	125 (39.8)	92 (29.3)	56 (17.8)	16 (5.1)				
		I know where to refer to if a homeless patient asks me about social support (314)	40 (12.7)	116 (36.9)	56 (17.8)	82 (26.1)	19 (6.1)				
Knowledge- patients	An awareness of the existence of something	Patients who are homeless know more than I do about coping with homelessness (314)	5 (1.6)	20 (6.4)	71 (22.6)	144 (45.9)	74 (23.6)	0.40	6.61	1.91	1.40
		Patients who are homeless know more than I do about general services available to them (315)	9 (2.9)	63 (20.0)	124 (39.4)	95 (30.2)	24 (7.6)				
Social/professional role and identity	A coherent set of behaviours and displayed personal qualities of an individual in a social or work setting	It is part of my role to ask homeless patients about their housing status (314)	46 (14.6)	112 (35.7)	115 (36.6)	36 (11.5)	5 (1.6)	0.60	14.79	9.46	3.08
		I should only initiate a conversation about homelessness if raised by the patient (314)	14 (4.5)	72 (22.9)	90 (28.7)	116 (36.9)	22 (7.0)				
		Only GPs or social care professionals should initiate a conversation about homelessness (314)	45 (14.3)	137 (43.6)	97 (30.9)	26 (8.3)	9 (2.9)				
		If I discuss aspects of homelessness with patients, I believe that I will be outside my own comfort zone (314)	20 (6.4)	71 (22.6)	84 (26.8)	119 (37.9)	20 (6.4)				
		Managing a homeless patient is compatible with my daily practice (311)	25 (8.0)	60 (19.3)	114 (36.7)	95 (30.5)	17 (5.5)				
Skills/belief about capabilities	An ability or proficiency acquired through practice	I am confident in my ability to advise appropriate medicines management strategies for homeless patients (315)	27 (8.6)	114 (36.2)	70 (22.2)	86 (27.3)	18 (5.7)	0.80	11.7	11.07	3.32
		I am confident in approaching social care services on behalf of homeless patients (314)	48 (15.3)	105 (33.4)	61 (19.4)	83 (26.4)	17 (5.4)				
		I am confident in my ability to advise homeless patients on aspects of self-care (314)	20 (6.4)	56 (17.8)	60 (19.1)	153 (48.7)	25 (8.0)				
		I am confident in my ability to identify patients who do not have a fixed abode (313)	30 (9.6)	105 (33.5)	85 (27.2)	77 (24.6)	16 (5.1)				
Optimism/pessimism	The confidence that things will happen for the best, or that desired goals will be attained	The NHS can do little to alleviate homelessness (314)	27 (8.6)	83 (26.4)	108 (34.4)	77 (24.5)	19 (6.1)	0.70	14.1	9.40	3.07
		Pharmacists can do little to alleviate the effects of homelessness (315)	16 (5.1)	101 (32.1)	97 (30.8)	81 (25.7)	20 (6.3)				
		Pharmacists can do little to alleviate the health impact of homelessness (314)	33 (10.5)	176 (56.1)	58 (18.5)	40 (12.7)	7 (2.2)				
		Patients who are homeless may not want my advice about housing or social support (314)	3 (1.0)	41 (13.1)	111 (35.4)	141 (44.9)	18 (5.7)				
		Managing homeless patients in pharmacy is pointless because they do not follow-up with their own care (315)	36 (11.4)	144 (45.7)	99 (31.4)	32 (10.2)	4 (1.3)				
Beliefs about consequences	Acceptance of the truth, reality, or validity about outcomes of a behaviour in a given situation	If I discuss aspects of homelessness with a patient, I believe that the patient may be reluctant to use my pharmacy again (313)	27 (8.4)	133 (42.5)	106 (33.9)	43 (13.7)	4 (1.3)	0.30	12.60	4.55	2.13
		Homeless patients will continue to have poor health outcomes regardless of pharmacy management (312)	15 (4.8)	129 (41.3)	67 (21.5)	86 (27.6)	15 (4.8)				
Goals	Mental representations of outcomes or end states that an individual wants to achieve	I would feel rewarded if I felt that a homeless patients’ health improved after I had provided advice to them (314)	2 (0.6)	3 (1.0)	24 (7.6)	156 (49.7)	129 (41.1)	0.43	7.98	1.90	1.38
		Pharmacy is not the right place to treat homeless patients (315)	66 (21.0)	133 (42.2)	76 (24.1)	31 (9.8)	9 (2.9)				
Intentions	A conscious decision to perform a behaviour or resolve to act or perform in a certain way	I would ask patients who are homeless whether they have access to food (314)	9 (2.9)	90 (28.7)	87 (27.7)	112 (35.7)	16 (5.1)	0.37	5.68	2.32	1.52
		Pharmacists should address medication needs and, not social circumstances of a patient (314)	18 (5.7)	119 (37.9)	98 (31.2)	65 (20.7)	14 (4.5)				
Environmental context and resources	Any circumstance of a person’s situation or environment that discourages or encourages the development of skills and abilities, independence, social competence, and adaptive behaviour	I have guidelines on advising homeless patients with no fixed abode (312)	117 (37.5)	135 (43.3)	47 (15.1)	11 (3.5)	2 (0.6)	0.70	16.7	15.71	3.96
		I have clear guidelines on how to advise homeless patients on their medicines use (313)	97 (31.0)	150 (47.9)	48 (15.3)	17 (5.4)	1 (0.3)				
		I have clear guidelines on how to advise homeless patients on accessing housing information (313)	121 (38.7)	141 (45.0)	39 (12.5)	11 (3.5)	1 (0.3)				
		Availability of guidelines on managing homeless patients in a community pharmacy will positively impact patient care (312)	14 (4.5)	20 (6.4)	57 (18.3)	163 (52.2)	58 (18.6)				
		I have had homeless patients complaining to me of stolen medicines (309)	80 (25.9)	95 (30.7)	60 (19.4)	55 (17.8)	19 (6.1)				
		I have had homeless patients complaining to me about lack of appropriate place to store their medicines (309)	80 (25.9)	109 (35.3)	72 (23.3)	37 (12.0)	11 (3.6)				
		I have sufficient time to discuss aspects of homelessness with my patients (314)	60 (19.1)	107 (34.1)	73 (23.2)	67 (21.3)	7 (2.2)				
Social influences	Those interpersonal processes that can cause an individual to change their thoughts, feelings, or behaviours	I have sufficient support from my colleagues in my pharmacy in managing homeless patients (313)	58 (18.5)	97 (31.0)	85 (27.2)	62 (19.8)	11 (3.5)	0.52	13.50	8.93	3.0
		Homeless patients often experience stigma from pharmacy staff (315)	26 (8.3)	80 (25.4)	105 (33.3)	80 (25.4)	24 (7.6)				
		Homeless patients often experience discrimination from pharmacy staff (314)	44 (14.0)	102 (32.5)	105 (33.4)	48 (15.3)	15 (4.8)				
		My organisation discourages me from discussing homelessness with a patient (311)	102 (32.8)	96 (30.9)	103 (33.1)	10 (3.2)	0 (0)				
		Individuals are responsible for their homelessness (313)	69 (22.0)	106 (33.9)	103 (32.9)	29 (9.3)	6 (1.9)				
Emotion	A complex reaction pattern, involving experiential, behavioural, and physiological elements, by which the individual attempts to deal with a personally significant matter or event	I become too emotional when I discuss aspects of homelessness with patients (315)	54 (17.1)	141 (44.8)	94 (29.8)	21 (6.7)	5 (1.6)	N/A	N/A	N/A	N/A

*N*/*A* not applicable

^a^Negative statements reversed scored for the analysis

### Perceived behavioural determinants in managing homeless patients

Responses in relation to the TDF statements are described below.

#### Knowledge, skills and resources in managing homeless patients

Respondents perceived a lack of knowledge and skills in identifying homeless patients and providing tailored advice and support. For example, less than a third of respondents (n = 93, 29.7%) agreed or strongly agreed that they were confident in their ability to identify patients who did not have a fixed abode (Table 2).

The majority of respondents indicated their awareness of the impact of homelessness on individual’s health and wellbeing, and medicines adherence (Table 2). However, only a third reported (n = 104, 33.0%) that they were confident in their ability to advise an appropriate medicines management strategy for people who are homeless. The majority agreed or strongly agreed (n = 218, 69.5%) that individuals facing homelessness had greater knowledge on coping mechanisms than respondents themselves.

The majority (n = 242, 94.5%) of respondents indicated not having covered the topic of homelessness during their undergraduate or postgraduate pharmacy training (n = 206, 97.2%), or during continuous professional development (CPD) (n = 225, 93.0%).

The majority reported that there was a lack of appropriate guidelines available for pharmacists to manage homeless patients (n = 299, 95.9%) or on how to offer tailored advice on medicines use (n = 295, 94.2%). It was perceived that availability of guidelines in managing homeless patients in community pharmacy would positively impact patient care (n = 221, 70.8%) (Table 2).

Most respondents perceived themselves to be less aware, less skilled or less able to refer homeless patients to social care services such as temporary accommodation providers. Less than a third (n = 101, 32.2%) indicated that they knew where to refer a patient if asked about social support.

#### Intentions

Less than a quarter (n = 53, 23.8%) would be willing to ask a homeless patient if they had a place to go for food or shelter. The reasons, as indicated in response to an open ended question, related to the perception that it would be ‘inappropriate’, perceived as ‘judgemental’ or ‘offensive’ to do so, a ‘difficult’ or ‘uncomfortable’ subject to raise, not having adequate knowledge on signposting and unsure of their wider circumstances.

#### Beliefs about consequences

Respondents indicated that pharmacists’ interventions can bring positive change in health outcomes. Most (n = 285, 90.1%) would feel rewarded in bringing about positive change amongst the homeless patients.

#### Social and professional role and identity

The majority (n = 273, 86.9%) disagreed or were unsure that it was their role to ask homeless patients about housing status. Approximately a quarter (n = 79, 25.2%), agreed or strongly agreed that pharmacists should address medication needs and not the social circumstances of a patient. Over two-fifths (n = 139, 44.3%) of respondents disclosed that broaching the subject of homelessness was outside their comfort zone.

### Social influences

Most respondents (n = 301, 63.7%) indicated that their workplace would support further involvement in care of the homeless patients. However, approximately a third (n = 106, 33.7%) and around a half (n = 146, 46.5%) of respondents indicated that homeless patients are likely to be facing stigma and discrimination respectively, from pharmacy staff (Table 2).

#### Optimism

Respondents indicated optimism that health care services, the healthcare professionals and pharmacists can do more to reduce homelessness and its health impact (Table 2).

The reliability analysis showed that the scales in relation to TDF domains knowledge (self), beliefs about capabilities (skills), optimism/pessimism, and environmental context and resources generated Cronbach’s alpha of ≥ 0.7 and hence were reliable.

### Objective knowledge assessment

Respondents scored highly in associating rough sleepers with homelessness, however, scored poorly on whether other vulnerably housed population fall under the definition of homelessness (Table 3). Only 4 (1.2%) respondents were able to correctly answer all questions in the knowledge assessment questions presented to them.

**Table 3:** Assessment of respondent knowledge about homelessness^a^

Knowledge assessment questions	Number of respondents (n)
What is the average years of death^b^ of a homeless individual in the UK? (n = 314)	
37 years	31 (9.9)
**47** **years**	**159 (50.6)**
57 years	89 (28.3)
58 years	35 (11.1)
Which of the below approximate number of rough sleeps in Scotland and England combined on any given night? (n = 312)	
**4000**	**42 (13.5)**
8000	85 (27.2)
10,000	125 (40.1)
12,000	60 (19.2)
An individual is only considered ‘homeless’ if he/she loses their home AND is living in a hostel? (n = 315)	
**Yes**	**209 (66.3)**
No	106 (33.7)
An individual is only considered ‘homeless’ if he/she loses their home AND is living in a bedroom at a friend’s house? (n = 315)	
**Yes**	**177 (56.2)**
No	138 (43.8)
An individual is only considered ‘homeless’ if he/she loses their home AND is living in a bedroom at a relative’s house? (n = 315)	
**Yes**	**171 (54.3)**
No	144 (45.7)
An individual is only considered ‘homeless’ if he/she loses their home AND is sleeping rough? (n = 315)	
**Yes**	**305 (96.8)**
No	10 (3.2)
An individual is only considered ‘homeless’ if he/she loses their home AND is sofa surfing with family and friends? (n = 315)	
**Yes**	**211 (67.0)**
No	104 (33.0)
An individual is only considered ‘homeless’ if he/she loses their home AND is all of above (315)	
**Yes**	**170 (54.0)**
No	145 (46.0)
Which group makes up the majority of the homeless population? (n = 310)	
Families with children	18 (5.8)
**Single men**	**286 (92.3)**
Single women	6 (1.9)
The number of homeless individuals in the UK is … (n = 313)	
Rising in number but very gradually	67 (21.4)
About the same for the last 10 years	19 (6.1)
Rising in number abruptly	57 (18.2)
**On the rise**	**170 (54.3)**

^a^Correct answer highlighted in bold

^b^Appeared as ‘life expectancy’ in the questionnaire which lends to a different meaning. This may have negatively impacted on the number of correct responses

### Positive and negative experiences of managing a homeless patient

A total of 171 and 169 respondents, via open ended questions, described a positive and negative experience respectively, they have had with a homeless patient.

### ‘Positive experiences’

Some respondents described offering free food, clothing and hygiene-related supplies to homeless persons presenting in pharmacy.

A homeless patient came in the pharmacy for his weekly medication pick up. He was pale and tired looking. He felt much better after we’ve given some snacks and water and he was grateful!

27 year old male pharmacist

Others described offering storage facilities for patient medicines and tailoring the dispensing services as per their needs.

Diabetic patient of NFA prescribed insulin - nowhere safe to store pens, constantly mislaid/stolen. Got Rx changed to weekly dispense supply kept in pharmacy fridge.

46 year old female pharmacist

Respondents also provided accounts of having seen the outcomes of the services they had offered to people who are homeless.

Discussing coming off both opioids and ‘‘legal highs’’ with a patient who had a lot of success in doing so alongside my support and is now in work.

28 year old male pharmacist

### Negative experiences

‘Negative experiences’ mostly related to perceived verbal and physical abuse, experiences of shoplifting, violence and aggression as well as intoxication and lack of hygiene.

Being shouted at and threatened numerous times usually by non-regular patients asking for a needle exchange. On one particular occasion, physical violence was threatened and police were called.

28 year old male pharmacist

Some respondents related such experiences to the wider social circumstances that homeless patients were facing.

Understandably many homeless patients feel threatened in their situation and often take their anger out on staff in pharmacies trying to help. I found on a regular basis the shouting from the patients was aggressive, rude and unnecessary.

46 year old female pharmacist

## Discussion

To the best of our knowledge, this is the first large scale survey investigating community pharmacists training, education and perceived behavioural determinants of management of homeless patients in the UK in their scope to reduce health inequality. Pharmacist respondents of this study indicated their willingness to offer support to the homeless population. Opportunities therefore exist to develop and implement knowledge and skills training programme for pharmacists in clinical areas often prevalent in homeless population. Pharmacists in the UK already offer some services that are relevant to the homeless population. Our previous research undertaking analysis of routinely collected data has indicated that over 50% of the patients registered with a primary healthcare centre for people who are homeless had been prescribed methadone treatment for opioid substitution [21]. In addition there is a higher rate of use of emergency care by the homeless [28]. Lack of adequate signposting has been reported to be one of the key barriers to access and use of primary and preventative health services by this population [29]. Results of this study suggest that pharmacists are willing to contribute to minimising inequality in health and access to healthcare.

Respondents of this study indicated that homelessness was not covered as part of their pharmacy undergraduate training and CPD sessions. The general pharmaceutical council, which regulates the initial education and training of pharmacists, indicates that pharmacy undergraduate students should be able to ‘collaborate with patients, the public and other healthcare professionals to improve patient outcomes’ as a key learning outcome [30]. There is a need to however, emphasise the care of the vulnerable population.

Despite willingness to offer greater involvement and support in helping people who are homeless, respondents of this study indicated lack of adequate knowledge and skills in broaching the subject of homelessness, identifying those suffering housing issues and proactively offering advice and support to the homeless patients.

Reluctance, or lack of knowledge, was reported in pharmacist’s ability to signpost homeless patients to social care services. Many respondents doubted whether it was in their remit to discuss aspects of patient housing and non-health related issues with homeless patients and many expressed low self-efficacy in broaching the subject of homelessness. There is an emphasis on greater involvement of healthcare professionals in the UK to be involved in joined up care including ‘social prescribing’ [31] activities and identifying vulnerable people at risk of being homeless early on [32]. Pharmacists involvement in social prescribing will allow them to refer patients to a named social care support worker in their local area when referring a homeless patient.

The results of this study suggest that community pharmacists may benefit from appropriate training and guidelines in offering proactive support and advice to homeless patients and in turn improving their confidence and skills. Establishing appropriate guidelines will enable community pharmacists to be aware of their remit in advising homeless patients on wider aspects of health and social care. Aspects of homelessness should be incorporated in undergraduate education and professional development trainings of community pharmacists to fill the gap in their knowledge and skills about the specialist needs of the homeless population.

### Study strengths and limitations

High standards of research methodology were applied including the development of a data collection tool informed by two previous qualitative research studies with the homeless population [20, 33] and the TDF; which was tested for face and content validity, and piloted prior to data collection. A further strength of the research was the wide expertise of the research team in the subject area including the involvement of healthcare professionals involved in provision of healthcare to people who are homeless.

Despite over 300 community pharmacists participating in this study, the response rate was low. This may be due to non-respondents’ lack of experience with providing pharmacy services to the homeless population or they may have been unaware of homeless patients use of their services. The study invitation and the participant information leaflet, however, had described that those with no experience of managing homeless respondents may also participate. Approximately a third of respondents’ pharmacies were based in the most deprived quintile zone of both England and Scotland which is proportionately a greater representation compared to other four deprivation zones. This may indicate that pharmacists in the most deprived regions have had greater involvement with homeless patients hence prompting their interest to participate in the study. Due to the low response rate caution should be exercised when seeking to generalise the findings. Respondent demography of our study shows slight under-representation of female respondents against national sample of pharmacists (49.5% study vs 59.4% nationally) [34].

We did not consider the inclusion three of the 14 behavioural TDF domains within the questionnaire: reinforcement, memory attention and decision process, and behavioural regulation [24] due to lack of adequate background information on the role of associated behavioural determinants. This may have limited our findings around other determinants influencing pharmacists’ practice. A small number of members in the questionnaire validation exercise is another study limitation.

## Future research

Future research should also incorporate the perspectives of pharmacists working in other healthcare sectors, pharmacy support staff and other healthcare and social workers around their views on greater involvement of pharmacists. There is a need to develop, implement, pilot and evaluate programmes that allows pharmacists to offer wider support to the homeless population including their involvement in social prescribing. Behaviour change technique taxonomy (BCTT version 1) provides a methodology for identifying content of complex behavioural change interventions that can be linked to the theory derived data from this research and the mechanism by which such interventions can improve practice [33]. Based on the findings of this study, the relevant interventions can be focused on the development, implementation and evaluation of evidence based guidelines and behavioural interventions to accommodate practicalities and pharmacists confidence, self-efficacy and intentions to provide care to the people who are homeless. General Pharmaceutical Council. Future pharmacists Standards for the initial education and training of pharmacists [35]. This research provides a base to consider both aspects. Research should also be conducted with homeless population on the barriers and opportunities for their use of pharmacy services.

## Conclusion

Community pharmacists in the UK may benefit from enhanced education, training, opportunities and guidelines that may positively impact on perceived behavioural determinants of their counselling and management of homeless patients such as knowledge, skills, confidence and intentions. Guidelines may enable community pharmacists to support homeless patients in alleviating the impact of homelessness, including pharmacist’s role in signposting to social services and providing tailored services. Aspects of homelessness should be incorporated in undergraduate education and professional development trainings of community pharmacists.
